# Exploring the structural, mechanical, magneto-electronic and thermophysical properties of *f* electron based XNpO_3_ perovskites (X = Na, Cs, Ca, Ra)

**DOI:** 10.1038/s41598-024-57341-2

**Published:** 2024-04-18

**Authors:** Sakshi Gautam, Dinesh C. Gupta

**Affiliations:** Condensed Matter Theory Group, School of Studies in Physics, Jiwaji University, Gwalior, Madhya Pradesh India

**Keywords:** Mechanical stability, Cohesive energy, Gruneisen parameter, Half-metallic, Materials science, Mathematics and computing

## Abstract

Here, we present systematic investigation of the structural and mechanical stability, electronic profile and thermophysical properties of *f*-electron based XNPO_3_ (X = Na, Cs, Ca, Ra) perovskites by first principles calculations. The structural optimization, tolerance factor criteria depicts the cubic structural stability of these alloys. Further, the stability of these materials is also determined by the cohesive and formation energy calculations along with mechanical stability criteria. The electronic structure is explored by calculating band structure and density of states which reveal the well-known half-metallic nature of the materials. Further, we have calculated different thermodynamic parameters including specific heat capacity, thermal expansion, Gruneisen parameter and their variation with temperature and pressure. The thermoelectric effectiveness of these materials is predicted in terms of Seebeck coefficient, electrical conductivity and power factor. All-inclusive we can say that calculated properties of these half-metallic materials extend their route in spintronics, thermoelectric and radioisotope generators device applications.

## Introduction

Over the last several decades, perovskite along with their derivatives such as halides, nitrides, etc. have been in-depth reviews owing to their applications in different domains like electronics, magnetics, optics, memory devices, etc.^1–4^. In view of this, the generic chemical formula of perovskites is ABX_3_ where A is a metal cation having 12-fold coordination while B is a metal cation having 6-fold coordination and X is a non-metallic anion^5^. Due to the flexibility and tunability of their crystal structures, they have tendency to show multidimensional features and have been widely anticipated for distinct applications. In this category, rare earth-based perovskites leap forward because of presence of *f-*states at the Fermi-level thus illustrate alluring electronic properties. This class of material exhibits a range of electrical properties because of their descripting the distinct electronic structures particularly ranging from metallic to insulating, or semiconducting and even half-metallic nature is preserved within these systems. However, in these kinds of electronic structures particularly, half-metallic materials have vast applications in spintronics which is considered as a new horizon of science exploiting the intrinsic spin in addition to its fundamental charge for multiple uses^6^. It has advantages over conventional electronics as it dissipates less heat and has faster processing speed. Besides, this kind of peculiarity these materials have been investigated either theoretically or experimentally for their commercial use in thermoelectric energy harvesting regimes where specifically the waste heat is directly converted into electrical energy by the principle of See beck effect^7,8^. Remarkably, these alloys pose their additional route towards the applicability’s in gas sensors, solid oxide fuel cells, solar cells, Lasers, LED etc.^9–13^. Further, rare earth-based oxides have also registered themselves to have relevant applications as catalyst, ionic conductors and optical materials^14,15^. Recently, a series of alloys based on *f-*electrons have been explored for different applications. Mudasir Younis et al. have scrutinized the various properties of RbMO_3_ (M = Np, Pu) perovskites and conclude the half-metallic nature of the alloys and address them as impactful thermoelectric materials^16^. Also, Nabi et al. have reported that BaBkO_3_ perovskite retains the half-metallic nature with high magnetic moment^17^. Besides this, properties of two actinide-based perovskites Ba_2_ErNbO_6_, Ba_2_TmNbO_6_ have been studied by Saveer Khandy et al. and predicted that the alloys are dynamically stable and retains the half-metallic nature influenced by their electronic structures and label them as smart materials^18^. Hence, through the literature survey we came up with the conclusion that *f*-electron-based alloys are apt for thermoelectric, spintronics and radio isotope generator applications. Moreover, because of their complex electronic structures, lanthanide actinide-based perovskites—particularly the neptunium oxides—may be the most promising members of the large perovskite family. Also, this element has interesting nuclear properties. Accordingly, in this present case of study we have figured out the structural, mechanical, electronic, magnetic, thermodynamic and thermoelectric properties of four perovskites based on rare earth element Neptunium specifically, XNpO_3_ (X = Na, Cs, Ca, Ra). The presence of Neptunium element in the studied perovskite system also open new possibilities for their applications in nuclear waste management also^19^. As, neither theoretical nor experimental information is yet available on these alloys so we are using first principle method.

## Computational approach

In this work all, the calculations have been performed by using full-potential linearized augmented plane wave (FP-LAPW) method as integrated in Wien*2K*^20^. To treat exchange and correlation a mix of approximations schemes like GGA^21^, GGA + U and GGA + mBJ^22^ have been inducted for this purpose. However, in case of many electron system the total wavefunction is represented by different basis set so, to locate the cut off parameter R_MT_ K_Max_ = 7.0 which was selected where R_MT_ is the smallest muffin tin radii and K_Max_ defines the expansion of plane wave. A mesh of 3000 K points was used in the momentum space and convergence criteria for energy and charge were set to ${10}^{-4}$ Ry and ${10}^{-4}$ eV respectively. Furthermore, to comprehend the mechanical strength we have specifically used the elastic code as incorporated in Wien2*k*^23^. Moreover, to characterize the thermal properties we have used the Gibbs2 package^24^ incorporated in Wien2*k*. To determine thermoelectric aspects of these alloys semi classical Boltzmann theory based Boltz Trap code is interfaced with WIEN2K code^25^.

## Results and discussion

### Structural properties

**Figure 1:**
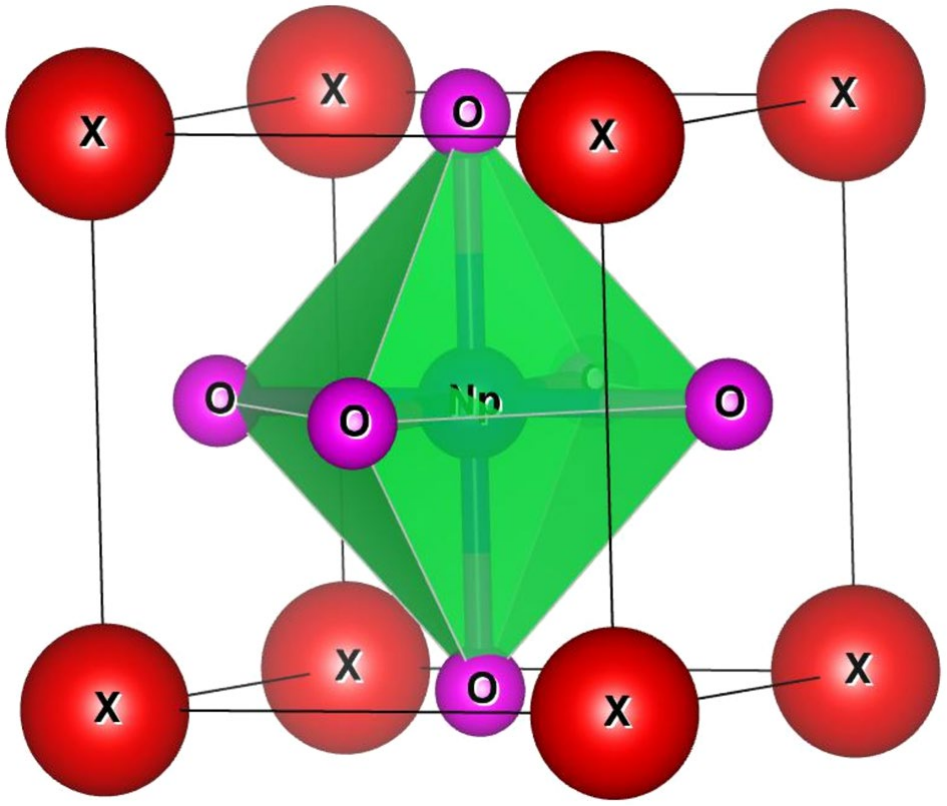
The crystal structure of XNpO_3_ perovskites.

**Figure 2:**
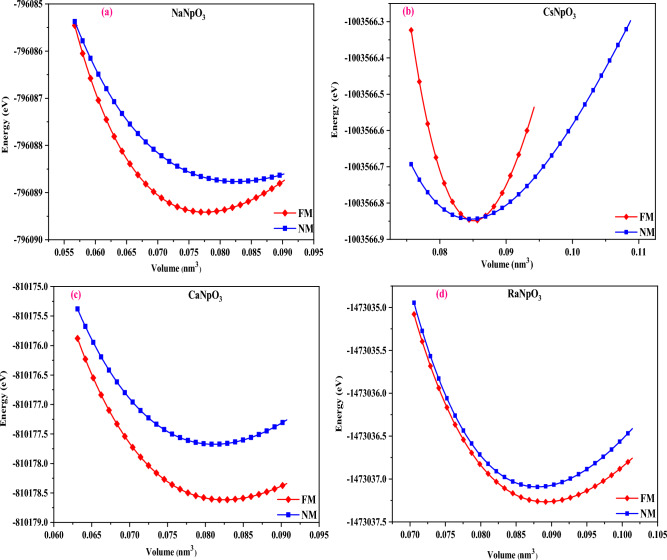
(**a**–**d**) Optimization plots of XNpO_3_ (M = Na, Cs, Ca, Ra) alloys in ferromagnetic and non-magnetic phases.

The opening move to study the behavior of any alloy is to apprehend the structural stability. So, to define the structure firstly, we have checked the stability of the alloys by calculating the tolerance factor^26^ which can be evaluated as *t* = 0.707 $(\frac{{r}_{A}+{r}_{O}}{{r}_{B}+{r}_{O}})$ where ${r}_{A}$ and ${r}_{B}$ be the radius of cations and ${r}_{O}$ is the radius of anion. The value of *t* lies within the range of 0.93–1.04 hence, ratify the cubic structure of these alloys^27^. The values of *t* are detailed in Table 1. The Wyckoff positions of atoms are descripted as X resides at (0, 0, 0) while Np at (0.5, 0.5, 0.5) and O at (0.5, 0.5, 0). The crystal structure of XNpO_3_ alloys is shown in Fig. 1. After that, we have determined the lattice constant with the help of relation $a_{0} = \alpha + \beta ({\text{r}}_{A} + r_{O} ) + \upgamma (r_{{\text{B}}} + {\text{r}}_{O} )$. Here, α = 0.06741, β = 0.4905, γ = 1.2921, while ${r}_{A}$ and ${r}_{B}$ are radii of cation, and ${r}_{O}$ is the radius of the anion^28^. By using the value of empirical lattice constant, we have checked the stability of the alloys in two different phases i.e. ferromagnetic and non-magnetic by performing energy volume optimisation using the Birch-Murnaghan equation of state^29^ and it was found that all the four alloys are stable in ferromagnetic phase as depicted in Fig. 2. The values of total ground state energy, lattice constant, unit cell volume, bulk modulus and derivative of bulk modulus obtained from the structural optimisation are illustrated in Table 1. Also, we have compared all these parameters with previously reported materials enlisted in Table 2 and on comparison we can say that our findings are comparable with others hence, justifying our results.

**Table 1:** Calculated lattice parameters (a_o_), minimum free energy (E_0_), bulk modulus (B), the derivative of bulk modulus (B^ʹ^_0_), Volume (V) and cohesive energy (eV/atom) for XNpO_3_ alloy (X: Na, Cs, Ca, Ra).

Parameters	NaNpO_3_	CsNpO_3_	CaNpO_3_	RaNpO_3_
a_o_ (nm)	0.44	0.44	0.43	0.45
a_o_ (A^0^)	4.4	4.4	4.3	4.5
E_0_ (eV) [FM]	− 796,089.41	− 1,003,566.84	− 810,178.52	− 1,473,037.31
E_0_ (eV) [NM]	− 796,088.76	− 1,003,566.12	− 810,177.56	− 1,473,036.13
B (GPa)	133.44	126.41	124.39	120.84
Bʹ_0_	4.27	4.81	4.08	4.30
Volume (nm^3^)	0.07	0.08	0.08	0.08
t	0.9	0.9	0.9	0.9
E_Coh_	4.62	4.35	4.95	4.19

**Table 2:** Calculated results of other perovskites for comparison.

Parameters	KNpO_3_	SrNpO_3_	RbNpO_3_	RbPuO_3_
a_o_ (A^0^)	2.80	4.40	4.33	4.32
E_0_ (eV) [FM]	− 808,049.44	− 878,164.37	− 872,767.84	− 894,867.97
B (GPa)	131.00	123.10	129.80	126.40
Bʹ_0_	4.37	4.07	4.55	4.73

Cohesive energy: We have also calculated the cohesive energy which is the energy required to separate the components from the crystal. It can be represented as: ${E}_{c}=\frac{\left[x{E}_{X}+y{E}_{Np}+z{E}_{O}\right]-{E}_{Total}}{x+y+z}$ ^30–33^. Here, ${E}_{Total}$ is the total optimized energy while ${E}_{X}$, ${E}_{Np}$ and ${E}_{O}$ are the energies of isolated X (Na, Cs, Ca, Ra), Np and O atoms, respectively and *x*, *y* and *z* are number of X, Np and O atoms respectively. The computed values of cohesive energy per atom are listed in same Table 1 which are positive for all the four materials. Hence, indicate that these alloys can be synthesized experimentally.

Formation energy: The energy designed to break the links among distinctive atoms in a crystal is known as formation energy, and has been calculated for the given materials using the relation $\Delta H={E}_{form}^{XNp{O}_{3}}= {E}_{total}^{XNp{O}_{3}}- (E$_X_$+ E$_Np_$+ 3E$_O_), where ${E}_{form}^{XNp{O}_{3}}$ conveys the optimum energy of the system; and $E$_X_, ${E}_{Np}$, $E$_O_ reflects the total energy of (Sodium, Caesium, Calcium, Radium), Neptunium and oxygen atoms in their stable elementary crystal structure. The $\Delta H$ obtained for NaNpO_3_, CsNpO_3_, CaNpO_3_ and RaNpO_3_ are (− 1.71, − 1.64, − 1.82 and − 1.54 eV) respectively^34^.

### Mechanical properties

The strength or capability of a material over the elimination of external forces executed on it can be determined by evaluating the set of Elastic constants. Total elastic constants are 81^35^. However, for cubic system only three elastic constants C_11_, C_12_, and C_44_ are enough to outline the elastic properties where the elastic constant C_11_ represents the longitudinal compression, C_12_ represents the transverse expansion while C_44_ denotes Shear Modulus, the calculated values of these constants are enlisted in Table 3. The stability of these compounds is again confirmed by the positive value of these constants. Also, the Born-Haung stability criteria [$${C }_{44}>0$$; $${C}_{11}+2{C}_{12}>0$$; $${C}_{11}-{C}_{12}>0$$] is followed^36^. Through the means of these constants we have calculated various other elastic parameters like Young’s, Bulk and shear moduli, etc. which are also detailed in Table 3. Firstly, we have estimated the bulk and shear modulii by Viogt-Reuss-Hill method^37^ then we have calculated one more important property i.e. Young’s modulus with the help of following relation $$Y=\frac{9BG}{3B+G}$$ which manifests the stiffness of the material. After that, we have also figured the different other parameters like poisson’s ratio (N), N = $$\frac{1}{2}\left(\frac{B-\frac{2 }{3}G}{B+\frac{1}{3}G}\right)$$, anisotropic factor (A), $$A=\frac{{2C}_{44}}{{C}_{11}-{C}_{12}}$$, Pugh’s ratio (B/G) and Cauchy’s pressure i.e. C″ = (C_12_ − C_44_) for these materials which are also listed is same Table 3. Poisson’s ratio indicates the degree of directionality of the covalent bonds its value is small (= 0.1) for covalent materials, while 0.25 for ionic materials. The values from Table 3, implied that the ionic bonding is dominating in these alloys^38^. For these alloys the calculated values are greater than 1 for Zener anisotropic factor hence, indicate the anisotropic nature^39^. To understand whether the material is ductile or brittle we have calculated the Pugh’s ratio of which values are greater than 1.75 for all the four perovskites so, suggesting the ductile nature which is further confirmed by Cauchy’s pressure as its value is positive for these materials^40^. Frantsevich et al. also suggest that if the value of Poisson’s ratio is greater than 0.26 then the material retain ductile nature so, again the ductile nature is established^41^. As, these materials are anisotropic in nature so, elastic waves have different velocities in different directions hence, we have figured out the magnitude of longitudinal ($${v}_{l}$$) and transverse waves ($${v}_{t1 }and {v}_{t2})$$ along (100), (110), (111) directions as enlisted in Table 4 by using Bugger’s relation^42^. Another, important property we have computed is Debye temperature $${\theta }_{D}$$ which associates the elastic and thermal properties, which we have computed with the help of mean sound velocity $${v}_{m}$$ calculated as: $${v}_{m}{=[\frac{1}{3}\left(\frac{2}{{v}_{l}3} +\frac{1}{{v}_{t}3}\right)]}^{\frac{-1}{3}}$$, where $$,{v}_{l}$$ and $${v}_{t}$$ are longitudinal and transverse sound velocities which can be find via Bulk and shear modulii by using Navier’s equation^43^ as given $${v}_{l}=\sqrt{\frac{3B+4G}{\rho }}$$ and $${v}_{t}=\sqrt{\frac{G}{\rho }}$$. The obtained values of longitudinal velocity, transverse velocity, mean velocity are listed in Table 5. The magnitude of $${\theta }_{D}$$ can be calculated as $${\theta }_{D}=\frac{h}{k}{\left(\frac{3n\rho {N}_{A}}{4\pi m}\right)}^\frac{1}{3}{v}_{m},$$ where h is Plank’s constant, k is Boltzmann’s constant, N_A_ is Avogadro number, ρ is density and $${v}_{m}$$ is average sound velocity. The calculated values of $${\theta }_{D}$$ for these alloys are also mentioned in Table 5. The high values specify that the melting point, hardness and thermal expansion coefficient of these materials should be extensive^44^. All the above calculated mechanical parameters provide a basis for predicting the performance and reliability of materials in real-world conditions.

**Table 3:** Calculated elasto-mechanical parameters at 0 GPa and 300 K. [Elastic constants C_11_, C_12,_ and C_44_ (GPa), shear modulus G (GPa), bulk modulus B (GPa); Young’s modulus Y (GPa) Poisson’s ratio N, B/G ratio, Cauchy’s pressure C'].

Alloy	C_11_	C_12_	C_44_	G	B	Y	Ν	A	B/G	C″
NaNpO_3_	289.06	56.01	38.36	61.02	133.69	158.90	0.30	0.32	2.19	17.65
CsNpO_3_	252.61	62.33	52.20	66.53	125.75	169.68	0.27	0.54	1.89	10.13
CaNpO_3_	310.63	84.84	27.96	50.96	160.10	138.22	0.35	0.24	3.14	56.88
RaNpO_3_	219.33	72.20	46.65	56.03	121.24	145.65	0.29	0.63	2.16	25.55

**Table 4:** Calculated sound (m/s) and averaged velocities along different directions.

Alloy	*v*_*l*_			$v_{{t_{1} }}$			$v_{{t_{2} }}$			$v_{m}v_{m}$			
Planes	[100]	[110]	[111]	[100]	[110]	[111]	[100]	[110]	[111]	[100]		[110]	[111]
NaNpO_3_	3310	2827	2647	1205	1205	1851	1205	1205	1851	1360		1596	1997
CsNpO_3_	2784	2536	2448	1265	1708	1574	1265	1265	1574	1417		1573	1717
CaNpO_3_	3445	2936	2746	1033	2077	1797	1033	1033	1797	1170		1406	1957
RaNpO_3_	2400	2248	2195	1107	1390	1302	1107	1107	1302	1239		1349	1433

**Table 5:** The calculated longitudinal, transverse, mean velocities (m/s) and Debye Temperature (K).

Alloy	*v*_t_	*v*_*l*_	*v*_*m*_	θ_D_
NaNpO_3_	1521	2855	1688	267
CsNpO_3_	1428	2565	1580	242
CaNpO_3_	1395	2952	1560	241
RaNpO_3_	1213	2269	1346	204

### Electronic properties

**Figure 3:**
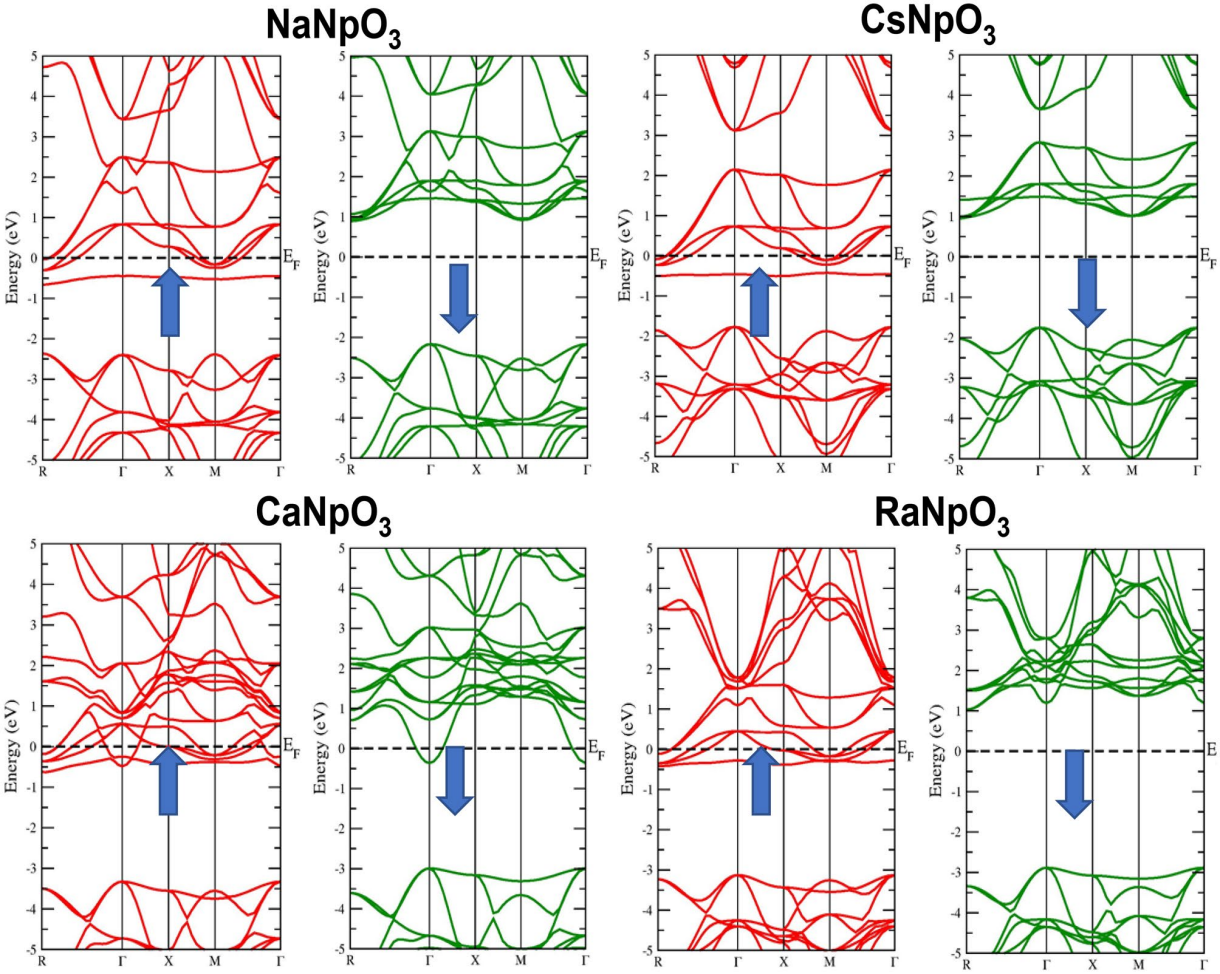
Band structure for XNpO_3_ (X = Na, Cs, Ca, Ra) alloys by GGA approximation (Red colour: Spin up and Green colour: Spin down).

**Figure 4:**
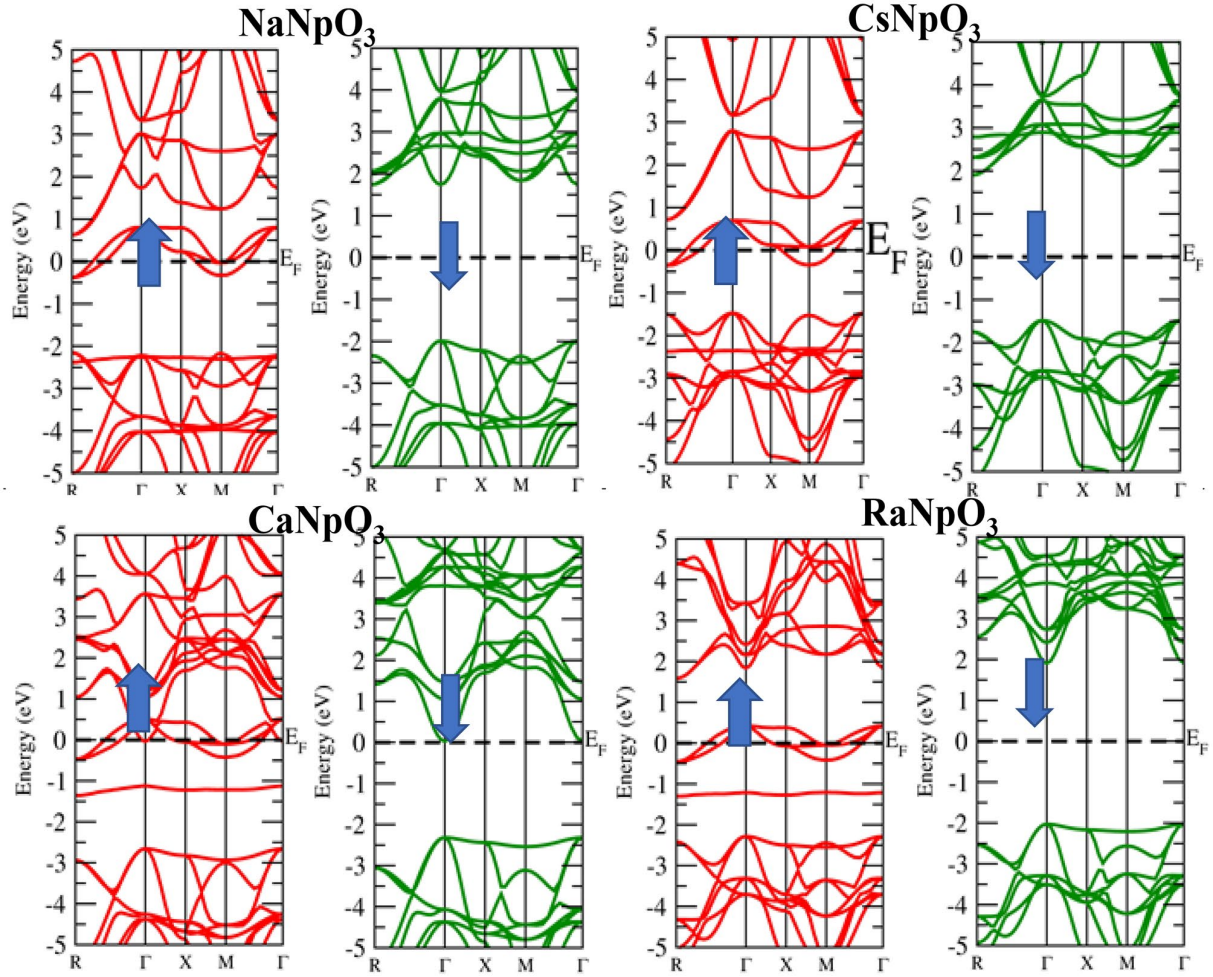
Band structure for XNpO_3_(X = Na, Cs, Ca, Ra) alloys by GGA + U approximation (Red colour: Spin up and Green colour: Spin down).

**Figure 5:**
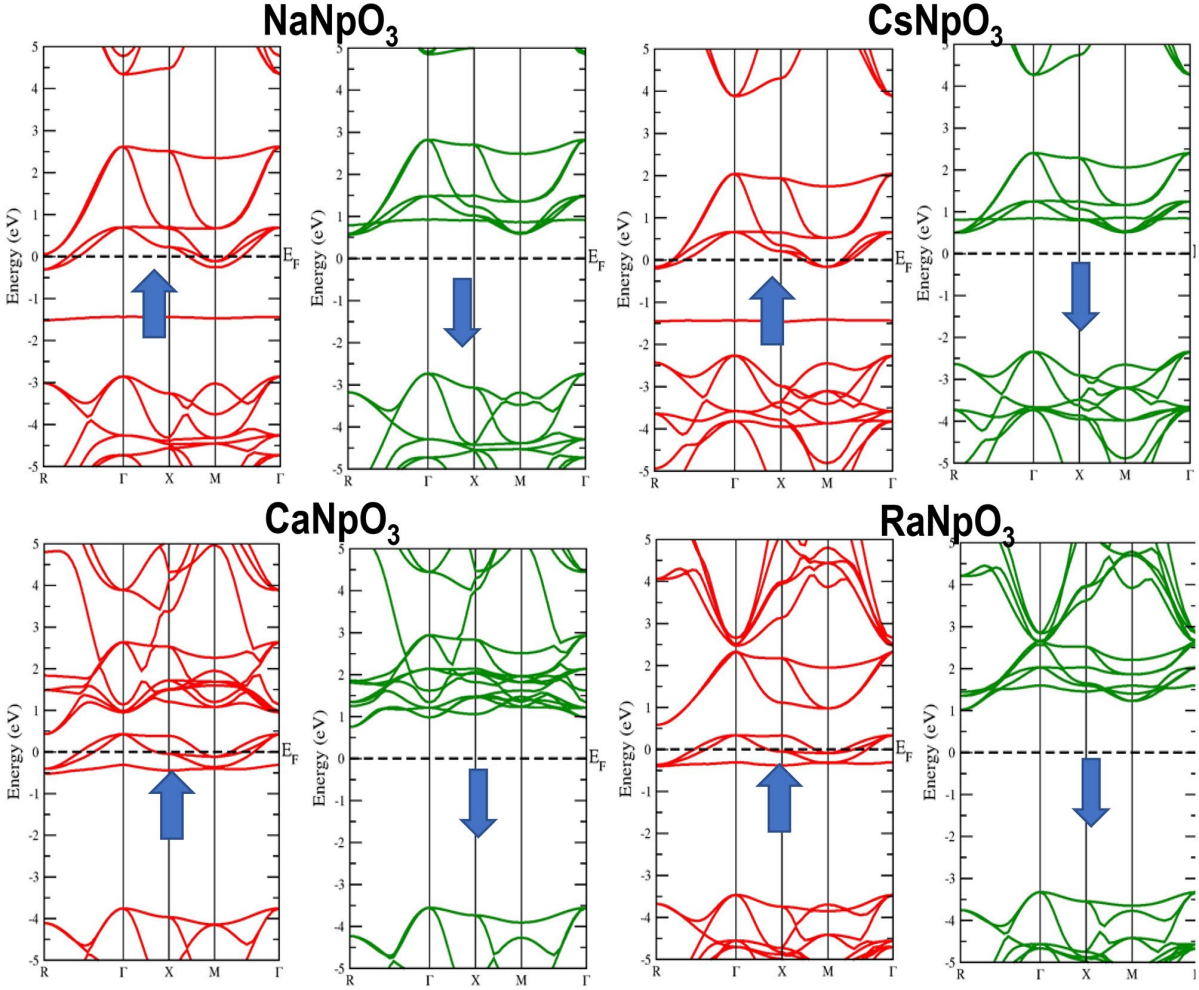
Band structure for XNpO_3_(X = Na, Cs, Ca, Ra) alloys by mBJ approximation (Red colour: Spin up and Green colour: Spin down).

**Figure 6:**
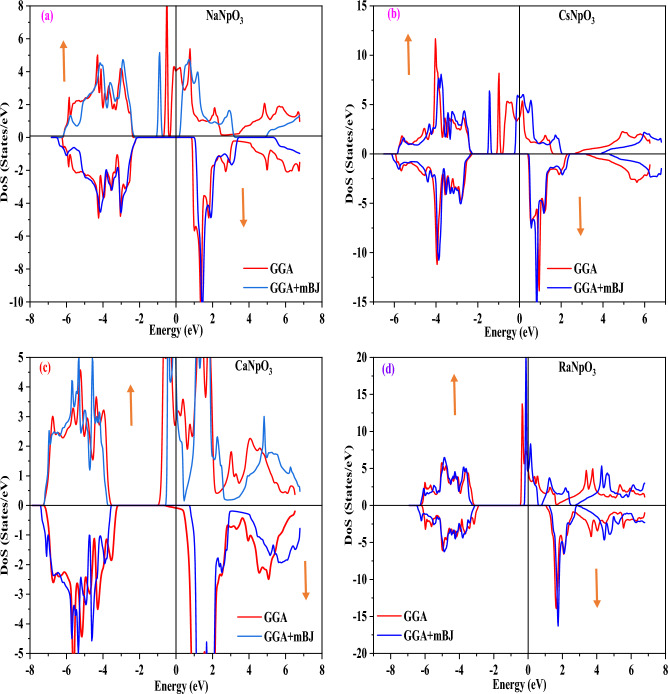
(**a**–**d**): Total density of states for XNpO_3_(X = Na, Cs, Ca, Ra) alloys.

**Figure 7:**
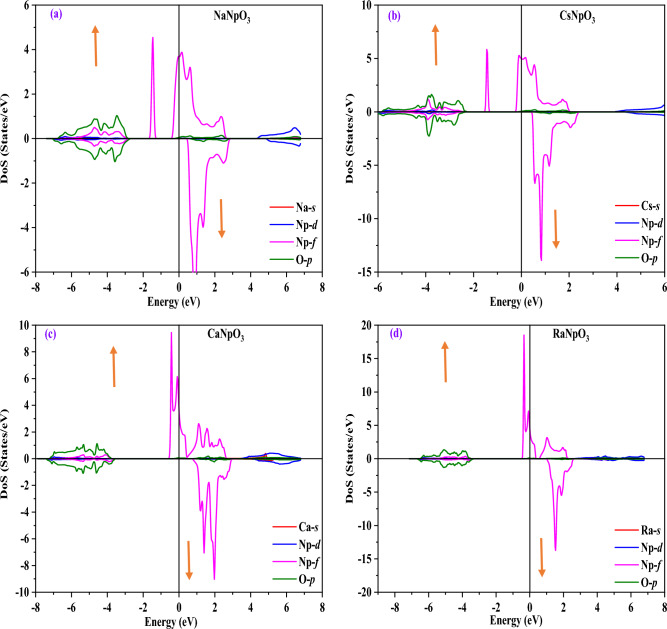
(**a**–**d**): partial density of states for XNpO_3_(X = Na, Cs, Ca, Ra) alloys.

To understand the applicability of material in different domains electronic properties play a significant role. The electronic properties can be discussed on the basis of band structure, total and partial density of states using spin polarized calculations. Firstly, we have examined the band structures of these alloys by using the values of optimized lattice constants and for this we have adopted GGA, GGA + U and GGA + mBJ approximations. The band structures by GGA approximation for these alloys are depicted in Fig. 3. From these pictures it is clear that for the spin up states these compounds depicts the metallic nature, while for spin down states three compounds namely NaNpO_3_, CsNpO_3_ and RaNpO_3_ shows the semiconducting nature while CaNpO_3_ depicts the metallic nature. The reason behind this is attributed due to the fact that *f* states arising from Np atom lie at fermi level in up spin giving rise to metallic nature while in a spin down there is a gap between two valence and conduction bands so giving rise to semiconducting nature for above mentioned three compounds. Whereas, for CaNpO_3_ the *f* states in both the spins are responsible for metallic character. The overall band structure of three alloys NaNpO_3_, CsNpO_3_ and RaNpO_3_ arrays the half-metallic nature with the band gap of (2.98, 2.64 and 3.98) eV respectively while metallic for CaNpO_3_. However, GGA is not sufficient to predict the clear-cut electronic structures of *d/f* electron-based systems because it underestimates the band gap values. So, to refine the electronic structure GGA + U is the next choice. Hence, we have employed the GGA + U where U = 0.29 Ry is the Hubbard potential. For GGA + U calculations, the energy bands cross the Fermi level presenting a metallic character in majority spin state, while in minority spin state semiconducting nature is reflected for NaNpO_3_, CsNpO_3_ and RaNpO_3_ whereas metallic character is retained in both up and down spins by CaNpO_3_ alloy as shown in Fig. 4. As GGA + U is semi-empirical in nature so we have employed GGA + mBJ potential. It proved to be a better choice because it can extract the screening parameter uniquely from electron density. The band structures by this potential along with high symmetric points of Brillouin zone are displayed in Fig. 5. On applying this potential, we observe that this potential had a significant effect on the conduction band in spin down channel the states shift towards higher energies, but in total these alloys feature the same half-metallic nature where the spin up pictures metallic nature while semiconducting nature is retained in spin down. The valence band maximum and conduction band minimum were found to be localized at Γ M symmetry points for NaNpO_3_, CsNpO_3_ and RaNpO_3_ with a band gap of (3.15, 2.73 and 4.35) eV respectively. Although, CaNpO_3_ shows the direct band gap of 4.44 eV. Owing to the appropriate band gap values these materials are apt for solar cells, photodetectors, LEDs and lasers^45^. Next, we have calculated density of states (total and partial) to understand the number of different states at a particular energy level. Figure 6. Represents the total density of states by GGA and GGA + mBJ simulation for XNpO_3_ alloys and from the graph we can see that all the four alloys reserve the same nature as evaluated by the band structures. To understand the projection of particular orbital of particular atom on the density of states we have computed the partial density of states by GGA + mBJ scheme as depicted in Fig. 7. As from the figure we inferred that there is no contribution of *s* orbital around the fermi level because of electropositive nature it loses its electron. The electronic configuration of Np is [Rn] *5f*^*4*^*6d*^*1*^*7s*^*2*^ Also, the *d*^1^-state of Np atom loses its electron to oxygen so there is no contribution of this state to the band formation as can be seen from PDOS plots of these alloys. So, here we can see the major peaks because of 5f state of Np atom. The peaks lie in the energy ranges of (− 7, − 6, 6.5 and − 6.2) eV for NaNpO_3_, CsNpO_3_, CaNpO_3_ and RaNpO_3_ respectively. In the up spin these states lie at the fermi level responsible for metallic character while in down channel these states are responsible for gap in between valence and conduction band hence, alloys behave as semiconductor in down channel. The overall behaviour we conclude is half-metallic for all the four alloys. As, no experimental data is reported for these materials so to verify the obtained results we have compared our findings with other reported materials of this class as illustrated in Table 7 and the comparison reveals that our results are appropriate.

### Magnetic properties

The electronic and magnetic properties are highly interdependent with each other. From the study of electronic properties especially from the density of states, it is clearly visualised that the Np *f* state is quite responsible to show the precise magnetic moment within these perovskite systems. Since the total magnetic moment is actually the sum of orbital and spin contributions but in our case study by the utilisation of different approximation schemes namely GGA, and GGA + mBJ as enlisted in Table 6 the sum of spin magnetic moment of these individual atoms of these alloys is considered give rise to magnetic moment. In contrast to the orbital contribution which is intrinsically being neglected due to the superior quench of highly correlated *d* and *f* states. The positive value of spin magnetic moment hints the ferromagnetic interaction caused by double exchange mechanism^46^ which is due to transfer of electrons from Np to O atom in our system. The calculated value of spin magnetic moments come out to be integer value which again support the half-metallic nature of the alloys^47^. The presence of spin magnetic moments displays the applications of these alloys in spintronics. The spin magnetic moments of XNpO_3_ alloys and other reported perovskites are in fair agreement as depicted from Tables 6 and 7.

**Table 6:** Magnetic moment in µ_B_ of the materials.

Materials	NaNpO_3_		CsNpO3		CaNpO3		RaNpO3	
Approximation	GGA	GGA + mBJ	GGA	GGA + mBJ	GGA	GGA + mBJ	GGA	GGA + mBJ
µ interstitial	0.31	0.29	0.22	0.21	0.51	0.43	0.43	0.35
µ_Na/Ra/Ca/Cs_	0.00	0.00	0.00	0.00	0.05	0.04	0.01	0.00
µ_Np_	1.93	1.89	2.10	2.02	2.64	2.62	2.77	2.73
µ_O_	0.08	0.06	0.11	0.07	0.07	0.04	0.07	0.03
Total	2.00	2.00	2.00	2.00	2.97	3.00	3.00	3.00

**Table 7:** Band gap (eV) and Magnetic moment in µ_B_ of the other reported materials.

Alloys	KNpO_3_	SrNpO_3_	BaNpO_3_
Band gap	3.13	4.59	3.80
µ interstitial	0.26	0.41	0.41
µ_K/Sr/Ba_	0.00	0.01	0.02
µ_Np_	1.93	2.67	2.76
µ_O_	0.00	0.03	0.08
Total	2.00	3.00	3.00
Nature of alloy	HM	HM	HM

### Thermodynamic properties

**Figure 8:**
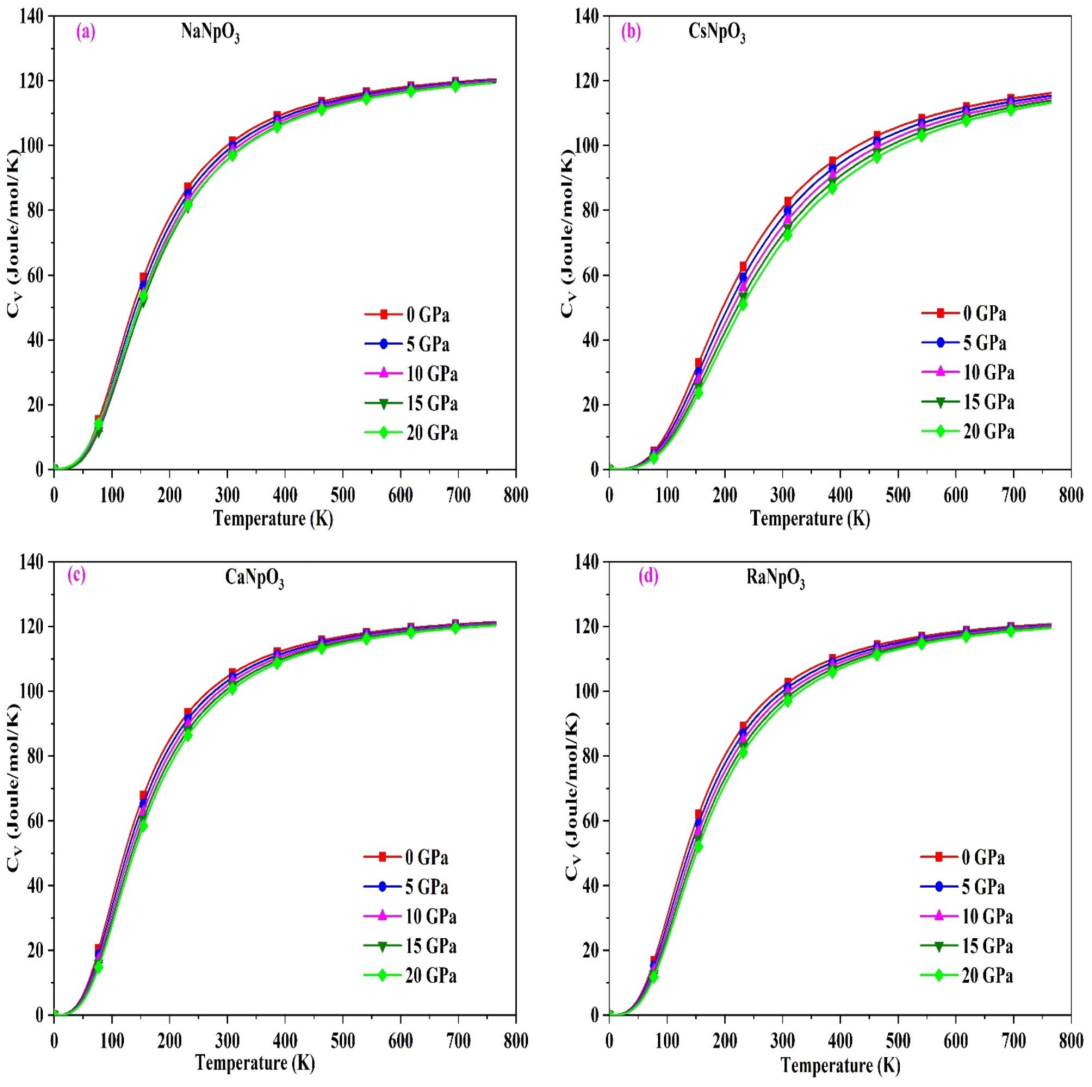
(**a**–**d**): The variation of specific heat C_V_ with temperature for XNpO_3_(M = Na, Cs, Ca, Ra) alloys.

For these alloys we have also calculated the thermodynamic properties under the function of temperature and pressure by using quasi-harmonic approximation which is based on quasi-harmonic Debye model^48^ within the temperature and pressure range 0–800 K;0–20 GPa. First of all, we have calculated the specific heat (${C}_{V}$), the variation with temperature at different pressure points is shown in Fig. 8. From the graph we can see that with increase in temperature the value of ${C}_{V}$ increases. Although, from the graph it can be seen that at lower temperatures variations are sharp obeying ${T}^{3}$ law^49^ whereas at higher temperature it follows the Dulong-petit law^50^. The underlying reason is that with increase in temperature atomic vibrations increases. Different from temperature, the pressure has very less effect on ${C}_{V}$. On increasing the pressure, there is a trivial decrease in the heat capacity as with increase in pressure there is a reduction in atomic vibrations. The value of ${C}_{V}$ for these alloys at room temperature are summarized in Table 8.

**Table 8:** The calculated value of specific heat (${C}_{V}$ in J/mol/K), Gruneisen parameter (γ), and Thermal expansion (α) in 10^–5^/K at room temperature.

Alloy	${C}_{V}$	γ	α
NaNpO_3_	100.26	1.90	1.56
CsNpO_3_	106.45	2.35	1.98
CaNpO_3_	102.73	1.84	1.60
RaNpO_3_	109.98	1.91	1.64

**Figure 9:**
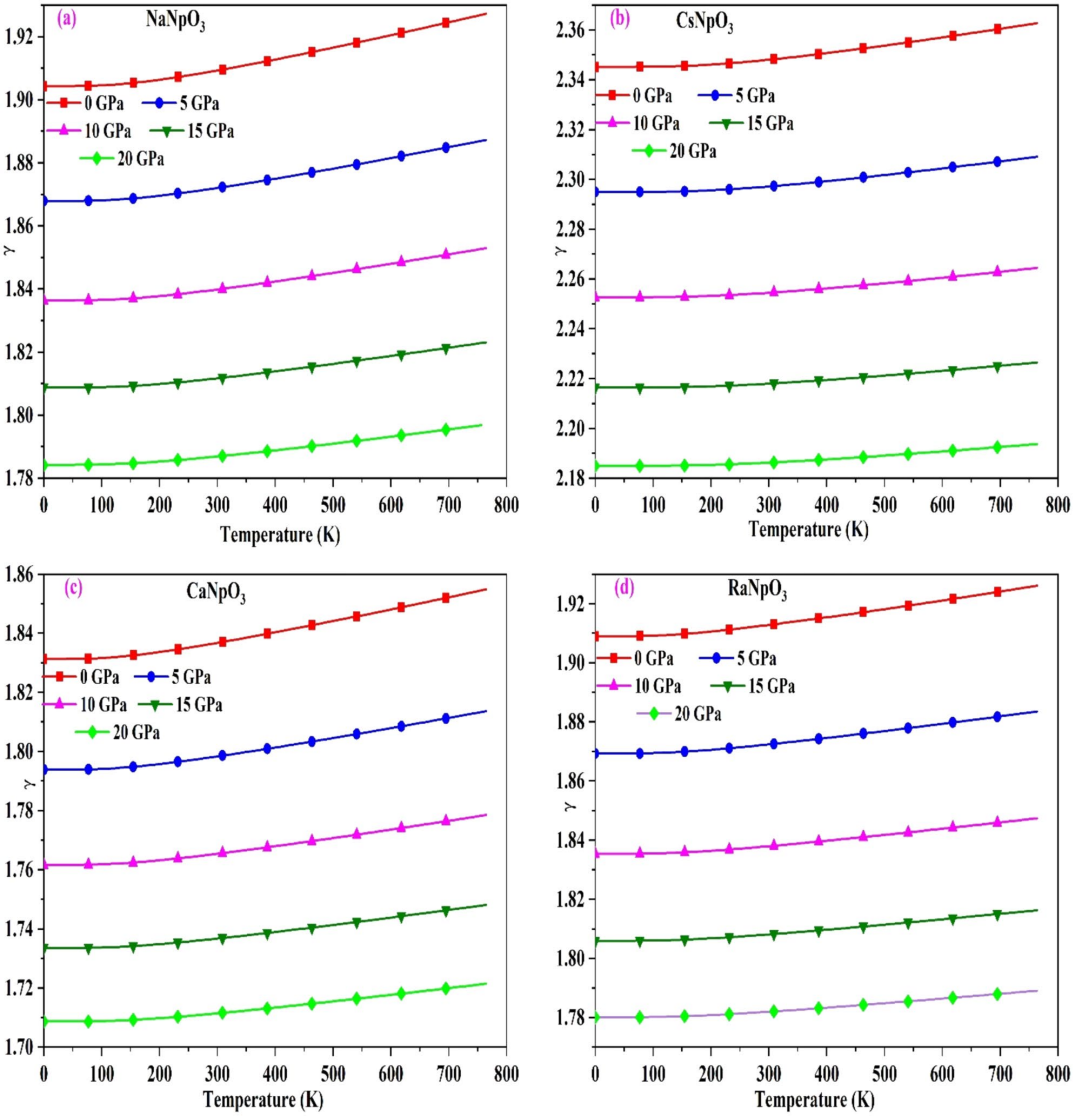
(**a**–**d**): The variation of Gruneisen parameter (γ) with temperature for XNpO_3_(X = Na, Cs, Ca, Ra) alloys.

After that, we have computed the Gruneisen parameter (γ) which assess the relation between thermal and elastic properties of solids as both the properties are controlled by same interatomic forces^51^. It explains the anharmonicity in the crystal. The variation is shown in Fig. 9, and from the displayed pictures we deduce that with increase in temperature it increases slightly, whereas, on increasing the pressure it decreases because of the drop in an harmonicity. So, we can say that pressure has strong effect on Gruneisen parameter. The values of γ for these alloys are mentioned in Table 8. Among these perovskites we can say that CsNpO_3_ shows better thermoelectric performance because of the greater an harmonicity.

**Figure 10:**
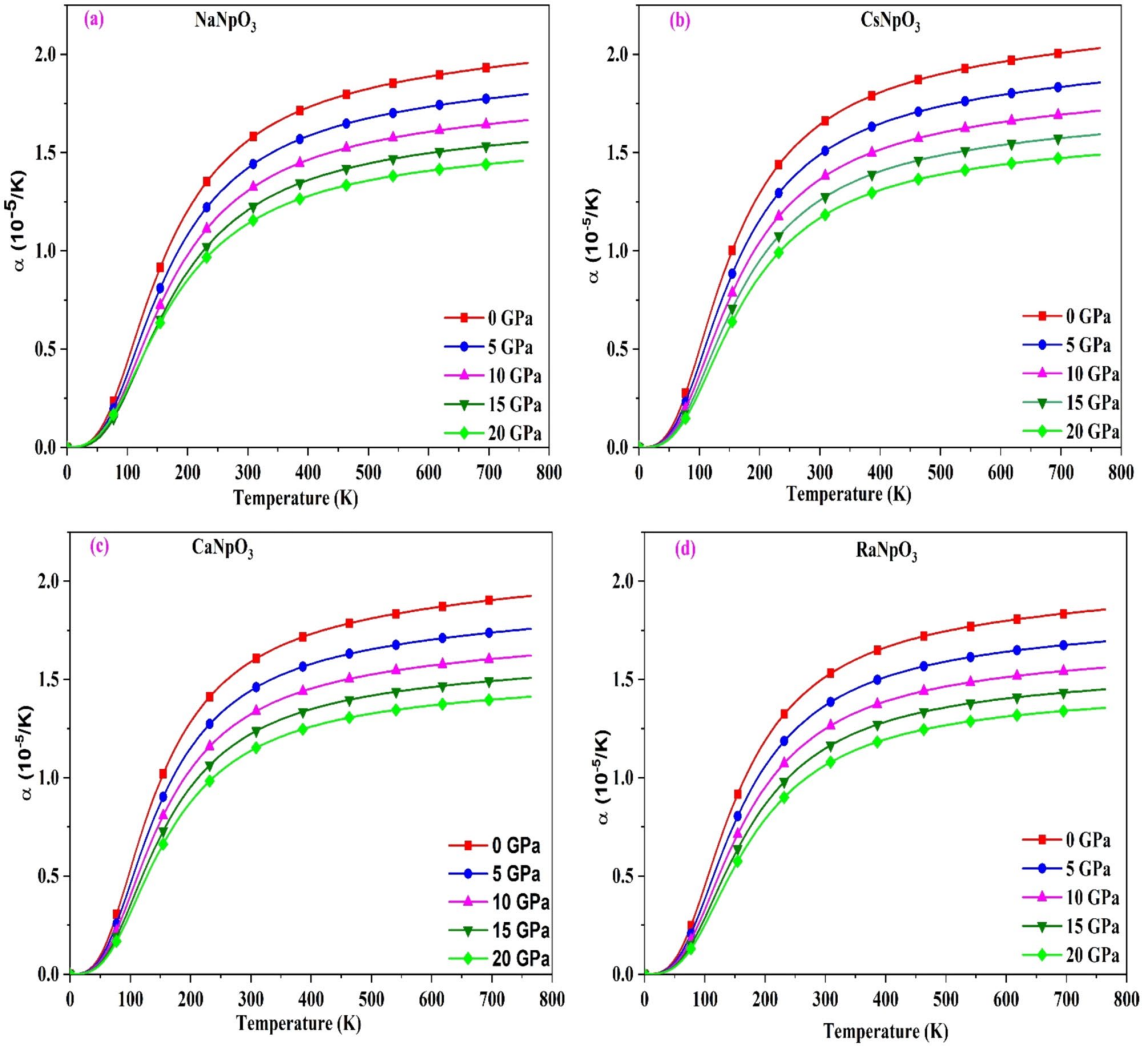
(**a**–**d**): The variation of thermal expansion (α) with temperature for XNpO_3_(X = Na, Cs, Ca, Ra) alloys.

Further, we have also calculated another important parameter i.e. the thermal expansion coefficient (α) enumerated as $\alpha =\gamma {C}_{V}/{B}_{T}V$. The plots for this parameter with temperature at different pressures are shown in Fig. 10. From the graph we can see that the trend of ${C}_{V}$ and α (Figs. 8 and 10 respectively) with temperature are similar, as $\gamma$ (Gruneisen parameter) and ${B}_{T}$ (Bulk modulus) are very less dependent on temperature. The behaviour of α with pressure are also sketched in the same Fig. 10. Wherefrom, we can observe that it decreases with increase in pressure as interatomic spacing decreases with the increase in pressure indicating strong bonding between the constituents. These outputs suggest that in thermal expansion pressure plays a notable role. Hence, while designing thermoelectric devices pressure effects should be considered. The values of α at room temperature are listed in Table 8.

### Thermoelectric properties

**Figure 11:**
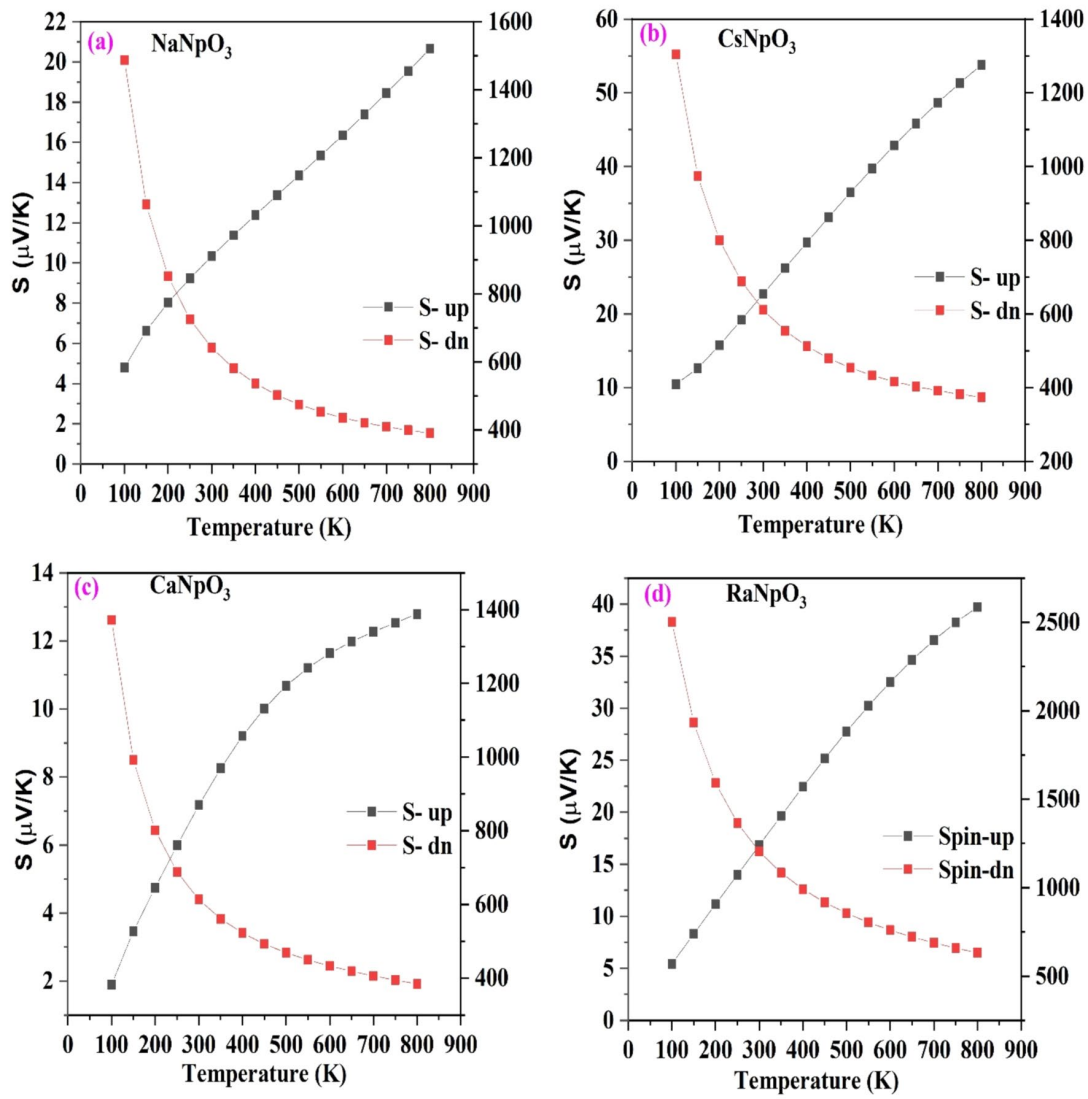
(**a**–**d**): Variation of Seebeck for both the spins against temperature for XNpO_3_(X = Na, Cs, Ca, Ra) alloys. [Left scale for spin up and right scale for spin down].

**Figure 12:**
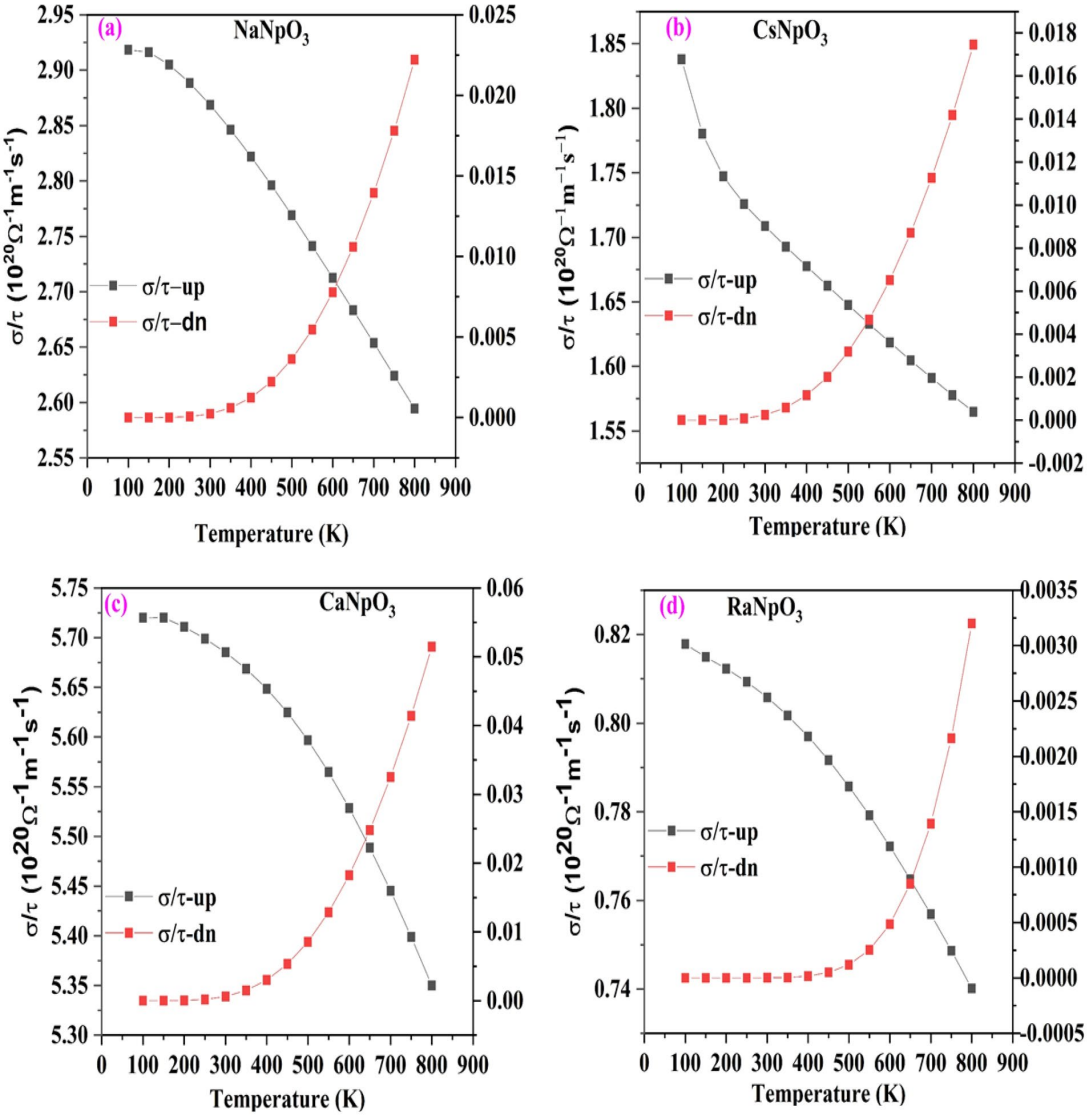
(**a**–**d**): Variation of electrical conductivity for both the spins against temperature for XNpO_3_(X = Na, Cs, Ca, Ra) alloys. [Left scale for spin up and right scale for spin down].

To examine the thermoelectric performance of these materials we have evaluated the different transport parameters i.e. Seebeck coefficient (S) electrical conductivity (σ/τ), thermal conductivity (κ) and power factor as a function of temperature within 0–800 K range. These coefficients are calculated precisely by using the Boltz Trap algorithm ingrained in Wein2K. To comment on the thermoelectric response of the material firstly, we have calculated the thermopower or Seebeck coefficient which can be symbolized as $S=\frac{8{\pi }^{2}{k}_{B}^{2}}{3e{\hslash }^{2}}{m}^{*}T{\left(\frac{\pi }{3n}\right)}^{2/3}{where, k}_{B}$, $\hslash$, ${m}^{*}$, and $n$ represent Boltzmann’s constant, reduced Planck’s constant, effective mass of carrier and number of carriers, respectively characterized in Fig. 11. From the graph we can see that the value of S for these materials in the spin-up channel increases with temperature, whereas in spin-down channel, its value decreases due to the reason that with increase in temperature carrier concentration increases thereby generating more electron hole pair which in turn leads to scattering effect responsible for a decrease in the Seebeck coefficient^30^. The value of the Seebeck coefficient for both the spins at 300 K (room temperature) and 800 K for all the materials is detailed in Table 9. Next, we have calculated the electrical conductivity which is highly related with the electronic structure of a material. Electrical conductivity as a function of temperature is picturised in Fig. 12 for both up and down spins. From the spin up graph it can be visualised that the electrical conductivity decreases with increase in temperature as with increase in temperature the vibrations increases which increase the mean free path resulting in the increase in resistance so, obstruct the flow of free electrons thus reducing the conductivity. On the other hand, the down spin graph of these alloys illustrates the semiconducting nature because with temperature electrical conductivity increases. The underlying reason is that in semiconductors at very low temperatures the donor atoms are not ionised but as the temperature rises ionisation process reach to maximum so all the donor atoms get ionised and at high temperature the excitations from the valence to conduction band are possible ensuing in the increase in electrical conductivity. Hence, overall these alloys limn the half-metallic nature which is agreeable to the electronic structure. The calculated value of electrical conductivity at room temperature and at maximum temperature for both the spins are listed in Table 10.

**Table 9:** The calculated value of the Seebeck coefficient (in microvolt/K) in both the spins for XNpO_3_ alloys.

Alloy	S (300 K)		S (800 K)	
	Spin up	Spin down	Spin up	Spin down
NaNpO_3_	10.33	641.06	20.66	390.52
CsNpO_3_	22.7	610.83	53.8	374.04
CaNpO_3_	7.16	613.53	12.79	384.59
RaNpO_3_	16.83	1204.57	39.72	632.58

**Table 10:** The calculated value of electrical conductivity in 10^20^ Ω^−1^ m^−1^ s^−1^ for both the spins.

Alloy	Spin state	(σ/τ) (300 K)	(σ/τ) (800 K)
NaNpO_3_	Up	2.86	2.59
	Down	0.0002	0.02
CsNpO_3_	Up	1.7	1.56
	Down	0.0003	0.01
CaNpO_3_	Up	5.68	5.34
	Down	0.0006	0.05
RaNpO_3_	Up	0.8	0.74
	Down	0.0009	0.003

**Figure 13:**
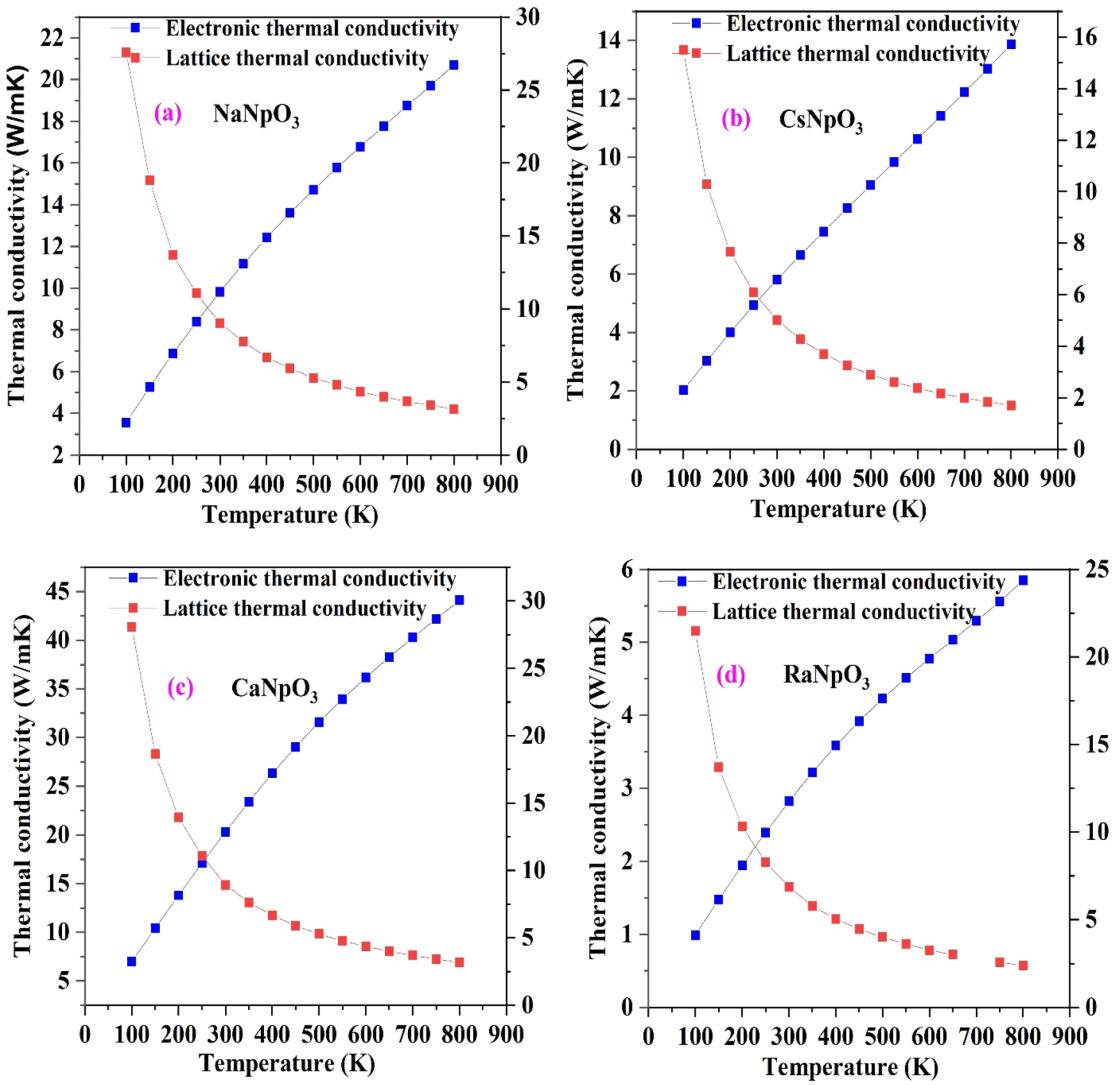
(**a**–**d**): Variation of thermal conductivity against temperature for XNpO_3_(X = Na, Cs, Ca, Ra) alloys. [Left scale for electronic thermal conductivity and right scale for lattice thermal conductivity].

In addition to above two parameters it is essential to calculate the thermal conductivity as to puzzle out how much heat is transported from the system, which contain two parts namely, electronic thermal conductivity (${\kappa }_{e}$) and lattice thermal conductivity (${\kappa }_{l}$). The electronic thermal conductivity can be directly obtained from the Boltz Trap code but this code is not capable to estimate the lattice part. So, to reckon this we have employed Slacks equation^52^. The variation of both parts with temperature is shown in Fig. 13 and from the graph it is reflected that electronic thermal conductivity increase with increase in temperature while, lattice thermal conductivity varies inversely with the temperature. The decaying behaviour of thermal conductivity strongly propose applications of these materials to utilised waste heat.

**Figure 14:**
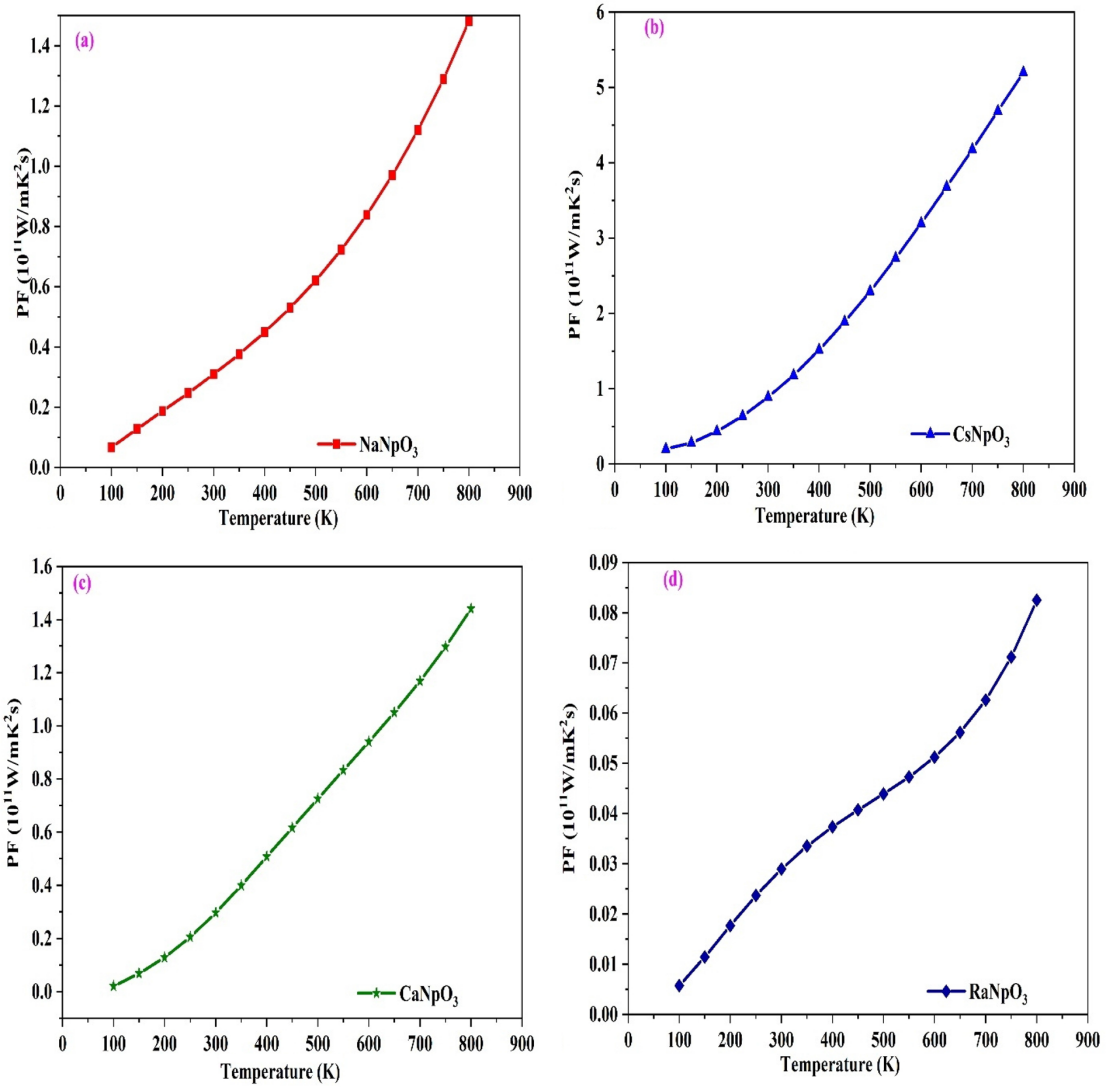
(**a**–**d**): Variation of Power factor against temperature for XNpO_3_(X = Na, Cs, Ca, Ra) alloys.

Thermoelectric performance of the material is determined by its power factor which depends on Seebeck coefficient and electrical conductivity. The power factor verses temperature graph is shown in Fig. 14, which shows a linear increase with temperature. It also gives information about the efficiency. Higher the power factor, more is the efficiency. It has maximum value at 800 K. The colossal power factor and decreasing trend of thermal conductivity favour these alloys to work as thermoelectric materials capable of being used to produce electricity and work as alternative energy sources. Also, the estimated values of these transport parameters are in line with the previously reported materials and other materials like CaMnO_3_, SrTiO_3_ which are commonly used in thermoelectric devices^53–56^. Also, we have compared results of our transport parameters with the skutterides and chalcogenides families which are highly efficient thermoelectric material and, on the study, we can say that XNpO_3_ alloys are also potential materials for thermoelectric applications.

## Conclusions

The structural optimization as well as cohesive and formasive energy calculations characterize the structural stability of the entitled perovskites. The elastic constants values further authenticate the mechanical stability of the materials. Our successful prediction of structural and mechanical stability would be helpful for the synthesis of these novel perovskites. The electronic profile reveals the half-metallic nature of the titled perovskites. For these alloys the value of spin magnetic moment is (2, 2, 3 and 3) μ_B_ for NaNpO_3_, CsNpO_3_, CaNpO_3_ and RaNpO_3_ respectively which is mainly due to Np atom. Also, the study of different thermodynamic parameters suggests that these oxide-based perovskites are applicable to a wide range of temperature and pressure. And lastly, the thermoelectric study reveals the decent values of different transport parameters of these alloys labelling them as potent thermoelectric materials for energy generation. Hence, on the whole we can say that the labelled alloys are suitable for energy production and solid-state device applications.

## Data Availability

The data would be available from the corresponding author on a reasonable request.
